# The Impact of SGLT1 Inhibition on Frailty and Sarcopenia: A Mediation Mendelian Randomization Study

**DOI:** 10.1002/jcsm.13614

**Published:** 2024-10-30

**Authors:** Bang‐Bang Huang, Yu‐Jie Zhang, Guang‐Feng Ruan, Xing Yu, Qin Liu, Mei‐Jin Zhang, Ming‐Zhong Yu, Ai Chen, Ye‐Bei Liang, Liang‐Di Xie, Li Luo

**Affiliations:** ^1^ Department of Geriatrics First Affiliated Hospital of Fujian Medical University, Institute of Neuroscience, Fujian Medical University Fuzhou China; ^2^ Fujian Hypertension Research Institute First Affiliated Hospital of Fujian Medical University Fuzhou China; ^3^ Department of Geriatrics, National Regional Medical Center, Binhai Campus of the First Affiliated Hospital Fujian Medical University Fuzhou China; ^4^ Clinical Research Center for Geriatric Hypertension Disease of Fujian Province First Affiliated Hospital of Fujian Medical University Fuzhou China; ^5^ Branch of National Clinical Research Center for Aging and Medicine First Affiliated Hospital of Fujian Medical University Fuzhou China; ^6^ Department of Geriatrics First Affiliated Hospital of Fujian Medical University, Fujian Key Laboratory of Molecular Neurology and Institute of Neuroscience, Fujian Medical University Fuzhou China; ^7^ Clinical Research Centre, Guangzhou First People's Hospital, School of Medicine South China University of Technology Guangzhou China; ^8^ Department of Epidemiology and Health Statistics, School of Public Health, Fujian Key Laboratory of Molecular Neurology and Institute of Neuroscience Fujian Medical University Fuzhou China; ^9^ Department of Cardiology First Affiliated Hospital, Fujian Medical University Fuzhou China; ^10^ Department of Cardiology, National Regional Medical Center, Binhai Campus of the First Affiliated Hospital Fujian Medical University Fuzhou China

**Keywords:** frailty, Mendelian randomization, sarcopenia, SGLT1 inhibition

## Abstract

**Background:**

Although pharmacological effects of SGLT2 inhibitors on the development of frailty and sarcopenia were known, the role of SGLT1 remained less clear. The present study investigated the possible effect of SGLT1 inhibition on these conditions and explored potential mediators.

**Methods:**

A two‐sample Mendelian randomization (MR) analysis was performed to assess the effect of SGLT1 inhibition on frailty index (FI) and low grip strength in individuals aged 60 years and older using both the FNIH and EWGSOP criteria. Subsequently, a two‐step MR analysis was conducted to investigate the mediating role of insulin resistance phenotype and identify potential mediators of the effect of SGLT1 inhibition on the FI and low grip strength from 1558 plasma proteins and 1352 metabolites.

**Results:**

Genetically predicted SGLT1 inhibition was associated with decreased FI (*β*: −0.290 [95% CI: −0.399, −0.181]) and reduced risk of low grip strength in individuals aged 60 years and older under both FNIH (*β*: −0.796 [95% CI: −1.216, −0.376]) and EWGSOP criteria (*β*: −0.287 [95% CI: −0.532, −0.041]). The two‐step MR analysis demonstrated the role of insulin resistance phenotype in mediating SGTL1 inhibition on alleviating frailty (mediation proportion = 19.56% [95% CI: 8.42%, 30.70%]). After screening, 24 proteins and 16 metabolites were identified as mediators of the impact of SGLT1 inhibition on FI. Additionally, 13 proteins and 16 metabolites were found to mediate the effect of SGLT1 inhibition on low grip strength according to FNIH criteria while 22 proteins and 6 metabolites were shown to mediate the impact of SGLT1 inhibition on low grip strength under EWGSOP criteria.

**Conclusions:**

SGLT1 inhibition potentially mitigated frailty and sarcopenia through several biological mediators, shedding new light for therapeutic intervention.

## 1 Introduction

The ageing population worldwide is placing an increasing strain on healthcare systems [1], leading to heightened awareness of age‐related issues, such as frailty and sarcopenia. Recent systematic reviews have reported that approximately 11% of elderly adults residing in the community experienced frailty [2] while the prevalence of sarcopenia was ranging from 9.9% to 18.6% according to different diagnostic criteria [3]. Therefore, investigating the potential pathological mechanisms of frailty and sarcopenia is of utmost importance for identifying novel and effective therapeutic targets for the elderly.

Studies have demonstrated that senior people with frailty had a higher risk of sarcopenia [4], and sarcopenia was associated with a greater risk of frailty in community‐dwelling elderly adults [5]. The crosstalk between frailty and sarcopenia probably attributed to the shared underlying pathophysiological mechanisms [6, 7, 8] in which insulin resistance in the skeletal muscle may be a key player. Indeed, some of the antihyperglycaemic medications have been reported to exhibit protective effects on skeletal muscle by alleviating the insulin resistance in diabetes [9]. A recent study reported that sotagliflozin, one of the newest antihyperglycaemic drugs inhibiting both sodium‐glucose co‐transporter 1 (SGLT1) and sodium‐glucose co‐transporter 2 (SGLT2), mitigated the impairment of viability, proliferation and migration abilities of skeletal muscle cells induced by high glucose and hypoxia [10]. However, whether the protective role of sotagliflozin on skeletal muscle resulted from the inhibition of SGLT1 or SGLT2 has not been fully elucidated. Previous studies have demonstrated the beneficial role of SGLT2 inhibition on skeletal muscle either in patients or animal models with frailty and sarcopenia [11, 12, 13, 14]. However, the impact of SGLT1, a related member of the SGLTs family, on frailty and sarcopenia still remained poorly understood.

Hence, the aim of this study was to explore the role of SGLT1 inhibition on frailty and sarcopenia and identify the potential mediators by using Mendelian randomization (MR) analysis.

## 2 Method

### 2.1 Study Design

**FIGURE 1:**
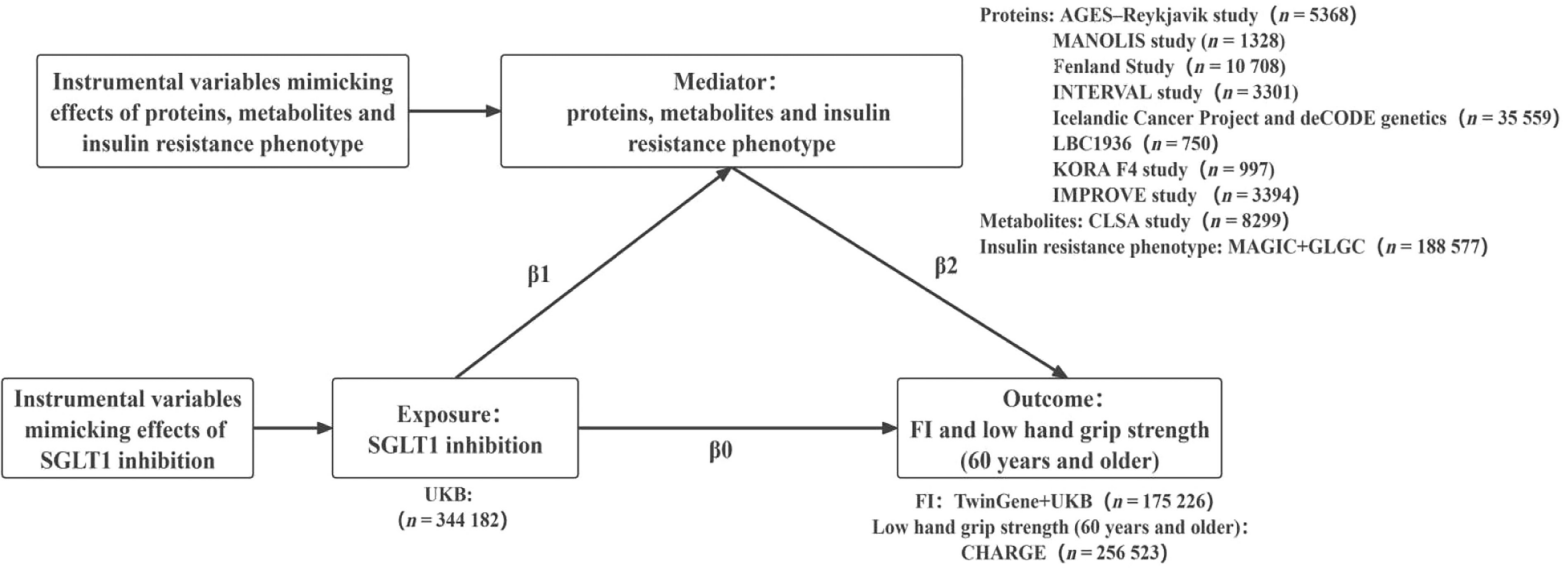
A flowchart illustrating the methodological framework and sources of GWAS databases utilized in the study. FI, frailty index; GWAS, genome‐wide association study; SGLT1, sodium‐glucose co‐transporter 1.

First, two‐sample MR analyses were conducted in the present study to assess the causal association between SGLT1 inhibition and frailty index (FI), as well as low grip strength in individuals aged 60 years and older. Second, genome‐wide association study (GWAS) statistics for 1558 proteins, 1352 metabolites and insulin resistance phenotype were utilized to identify the potential targets that were associated with FI and low grip strength. After screening, the casual associations between SGLT1 inhibition and the potential targets were evaluated. Finally, the delta method [15] was employed to estimate the mediating effect among the targets showing causal associations with both SGLT1 inhibition and outcomes (Figure 1).

### 2.2 Instrumental Variables for SGLT1 Inhibition

Candidate instrumental variables were screened and filtered through a multi‐step process in primary analysis. First, genetic variants linked to mRNA expression levels of SLC5A1, known as the gene of SGLT1, were identified by using data from the Genotype‐Tissue Expression (GTEx) [16]. Second, selected variants were further refined to identify the SNPs associated with the glycated haemoglobin (HbA1c), a supposed indicator of SGLT1 inhibitor for its glucose‐lowering effect [17, 18], at a statistical significance threshold of 1 × 10^−4^. GWAS data for HbA1c based on an unrelated group of European individuals without diabetes in the UK Biobank were used [19] (Table S1). Subsequently, SNPs in linkage disequilibrium (LD) of *r* ^2^ > 0.8 within 250 kb were removed, according to the 1000 Genomes European reference panel [19]. Finally, the statistical power of these variants were estimated by *F* statistics, where *F* > 10 indicated a low probability for weak instrument bias [20]. Consequently, 16 SNPs were selected to mimic the effect of SGLT1 inhibition (Table S2). Similarly, the instrumental variables for insulin resistance phenotype, plasma proteins and metabolites were also identified (Method S1, Table S3).

### 2.3 Study Outcomes

In the present study, FI was utilized as an indicator of frailty severity [21]. The GWAS summary data of FI were derived from participants of the UK Biobank (*n* = 164 610, aged 60–70 years and 51.3% females) and TwinGene (*n* = 10 616, aged 41–87 years and 52.5% females), where the FI was calculated based on self‐reported items (49 items in UK Biobank, 44 items in TwinGene) on symptoms, disabilities and diagnosed diseases and presented as a proportion of the sum of all deficits (Table S4) [22]. Additionally, the diagnosis of low grip strength, a key characteristic of sarcopenia, was referred to the criteria established by the Foundation for the National Institutes of Health (FNIH) [23] and the European Working Group on Sarcopenia in Older People (EWGSOP) [24], respectively. The GWAS statistics of low grip strength in individuals aged 60 years and older under both FNIH (grip strength: < 26 kg male; < 16 kg female) and EWGSOP (grip strength: < 30 kg male; < 20 kg female) criteria was from the CHARGE study, including 256 523 individuals of European descent [25] (Table S1).

### 2.4 Statistical Analysis

Two‐sample MR analyses were performed to investigate the effect of SGLT1 inhibition on frailty and low grip strength (β0). In the analysis, the inverse variant weighted (IVW) method [26] was used as the primary analytical approach, offering the most efficient and powerful estimates. Mendelian Randomization Pleiotropy RESidual Sum and Outlier (MR‐PRESSO) [27] was employed to identify and correct potential horizontal pleiotropy and heterogeneity by removing outlier SNPs. Moreover, MR‐PRESSO also provided the casual estimation of the MR analysis. The Cochran's *Q* statistic in IVW and MR‐Egger were used to assess the presence of heterogeneity in results [28]. In addition, two‐step MR analyses were used to identify the potential mediators (Method S2). Furthermore, sensitivity analyses were performed to validate the stability of the results (Method S3, Table S5).

## 3 Results

### 3.1 SGLT1 Inhibition Attenuated Frailty and Low Grip Strength

**FIGURE 2:**
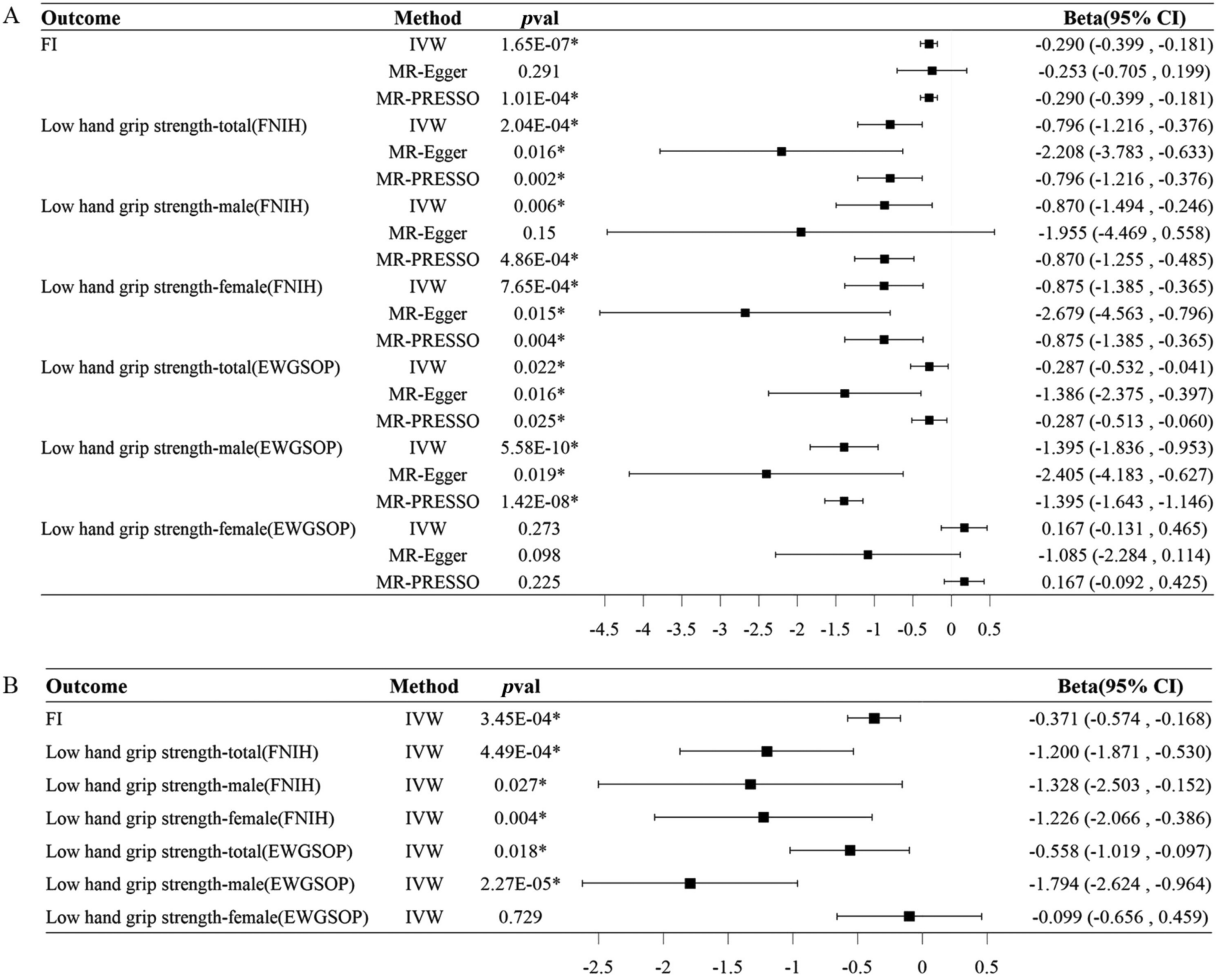
Causal effect of SGLT1 inhibition on FI, as well as low grip strength in both male and female individuals aged 60 years and older under both FNIH and EWGSOP criteria. (A) In the primary analysis, genetic variants demonstrating significant associations with both HbA1c levels and mRNA expression of the SLC5A1 gene were identified as instrumental variables for MR analysis to investigate the causal relationship between SGLT1 inhibition and outcomes. (B) In the validation analysis, functionally damaging missense variants within the SLC5A1 gene, previously documented in literature, were employed to mimic the effect of SGLT1 inhibition in the MR analysis. CI, confidence interval; EWGSOP, European Working Group on Sarcopenia in Older People; FI, frailty index; FNIH, Foundation for the National Institutes of Health; IVW, inverse variance weighted; MR, Mendelian randomization; *p*val, *p* value; SGLT1, sodium‐glucose co‐transporter 1.

In the primary analysis, genetic variants associated with both HbA1c levels and mRNA expression of the SLC5A1 gene were identified as instrumental variables for MR analysis. The results of the IVW method indicated that SGLT1 inhibition was associated with decreased FI (*β*: −0.290 [95% CI: −0.399, −0.181]) and reduced risk of low grip strength in individuals aged 60 years and older under both FNIH criteria (*β*: −0.796 [95% CI: −1.216, −0.376]) and EWGSOP criteria (*β*: −0.287 [95% CI: −0.532, −0.041]). Furthermore, a similar trend was observed in the association between SGLT1 inhibition and low grip strength across both genders. Under FNIH criteria, SGLT1 inhibition was associated with a decreased risk of low grip strength in both male (*β*: −0.870 [95% CI: −1.494, −0.246]) and female (*β*: −0.875 [95% CI: −1.385, −0.365]) individuals aged 60 years and older. For EWGSOP criteria, statistical significance was observed in the relationship between SGLT1 inhibition and low grip strength in males (*β*: −1.395 [95% CI: −1.836, −0.953]), whereas no significant relationship was found in females (*β*: 0.167 [95% CI: −0.131, 0.465]). In addition, the directions of the MR‐Egger and MR‐PRESSO method estimates were consistent with those of IVW method (Figure 2A). Sensitivity analysis indicated that there was no evidence of heterogeneity in the results based on Cochran's *Q* statistic in the IVW and MR‐Egger methods. Moreover, the intercept in MR‐Egger and global test in MR‐PRESSO suggested that horizontal pleiotropy did not impact the results (Table S6). In the validation analysis, genetic variants previously reported to represent the loss‐of‐function of SLC5A1 gene were employed to mimic the effect of SGLT1 inhibition, and consistent results were observed (Figure 2B, Table S7).

### 3.2 SGLT1 Inhibition Ameliorated Frailty Through Alleviating Insulin Resistance

First, we assessed the impact of the insulin resistance phenotype on FI. Results in the IVW method revealed a robust causal association between insulin resistance and FI (*β*: 0.274 [95% CI: 0.163, 0.386]). After removing outlier SNPs, there was no evidence of heterogeneity and pleiotropy (Table S8). However, insulin resistance phenotype turned out to be unrelated with low grip strength under both FNIH (*β*: −0.139 [95% CI: −0.538, 0.259]) and EWGSOP (*β*: −0.218 [95% CI: −0.479, 0.044]) criteria. Next, we further estimated the effect of SGLT1 inhibition on insulin resistance phenotype and found out a significant casual association (*β*: −0.207 [95% CI: −0.289, −0.125]). Subsequently, the two‐step MR analysis was conducted to assess the role of insulin resistance in mediating the causal effect of SGLT1 on FI. As shown in Table S9, insulin resistance accounted for 19.56% (95% CI: 8.42%, 30.70%) of the total effect of SGLT1 inhibition on FI.

### 3.3 Mediation MR of SGLT1 Inhibition, Plasma Proteins and FI as Well as Low Grip Strength

**FIGURE 3:**
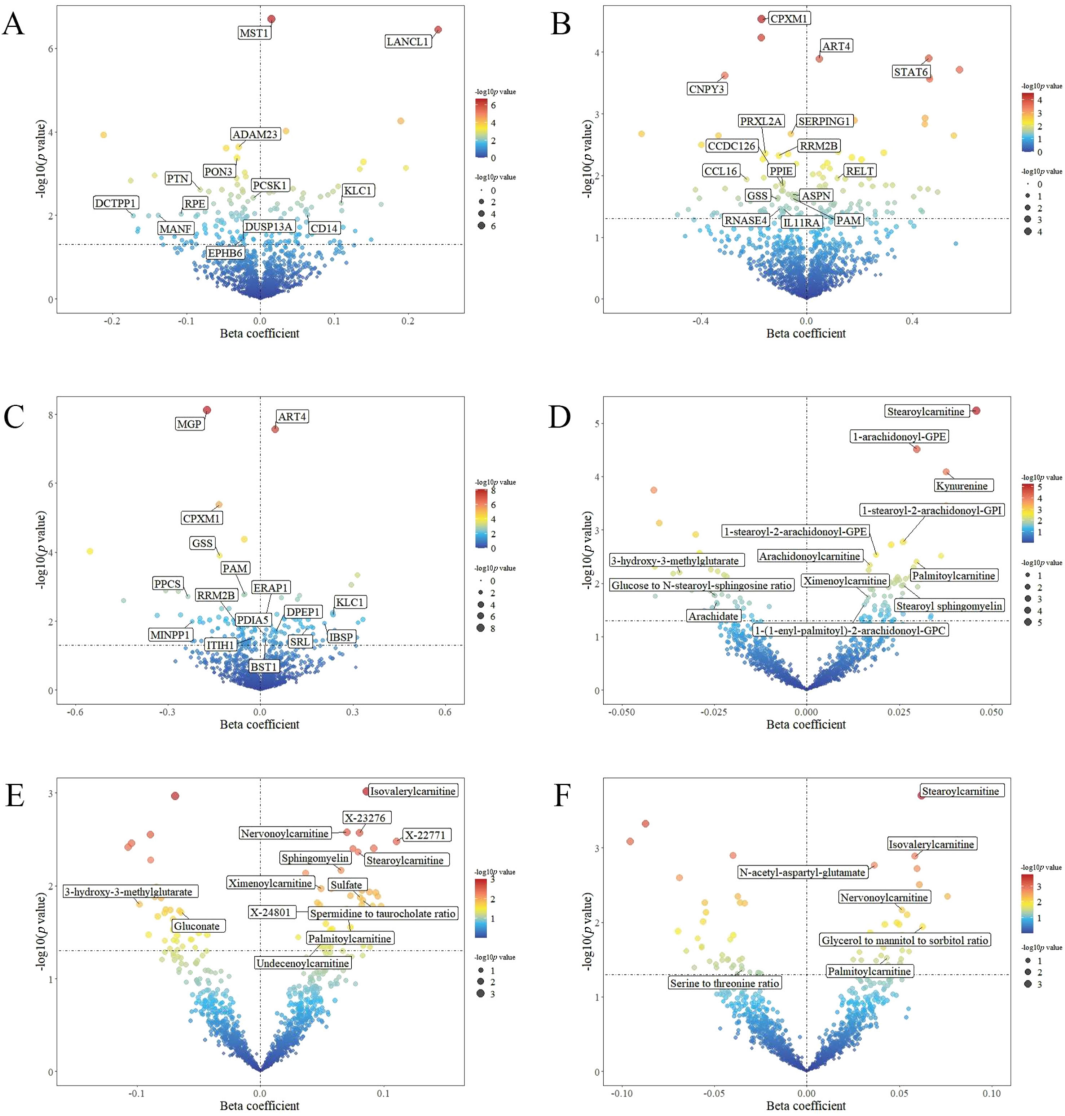
Causal effect of plasma proteins and metabolites on FI, as well as low grip strength aged 60 years and older under both FNIH and EWGSOP criteria. (A) Volcano gram illustrating the association between plasma proteins and FI. (B) Volcano gram illustrating the association between plasma proteins and low grip strength (60 years and older) under EWGSOP criteria. (C) Volcano gram illustrating the association between plasma proteins and low grip strength (60 years and older) under FNIH criteria. (D) Volcano gram illustrating the association between plasma metabolites and FI. (E) Volcano gram illustrating the association between plasma metabolites and low grip strength (60 years and older) under EWGSOP criteria. (F) Volcano gram illustrating the association between plasma metabolites and low grip strength (60 years and older) under FNIH criteria. EWGSOP, European Working Group on Sarcopenia in Older People; FI, frailty index; FNIH, Foundation for the National Institutes of Health.

**FIGURE 4:**
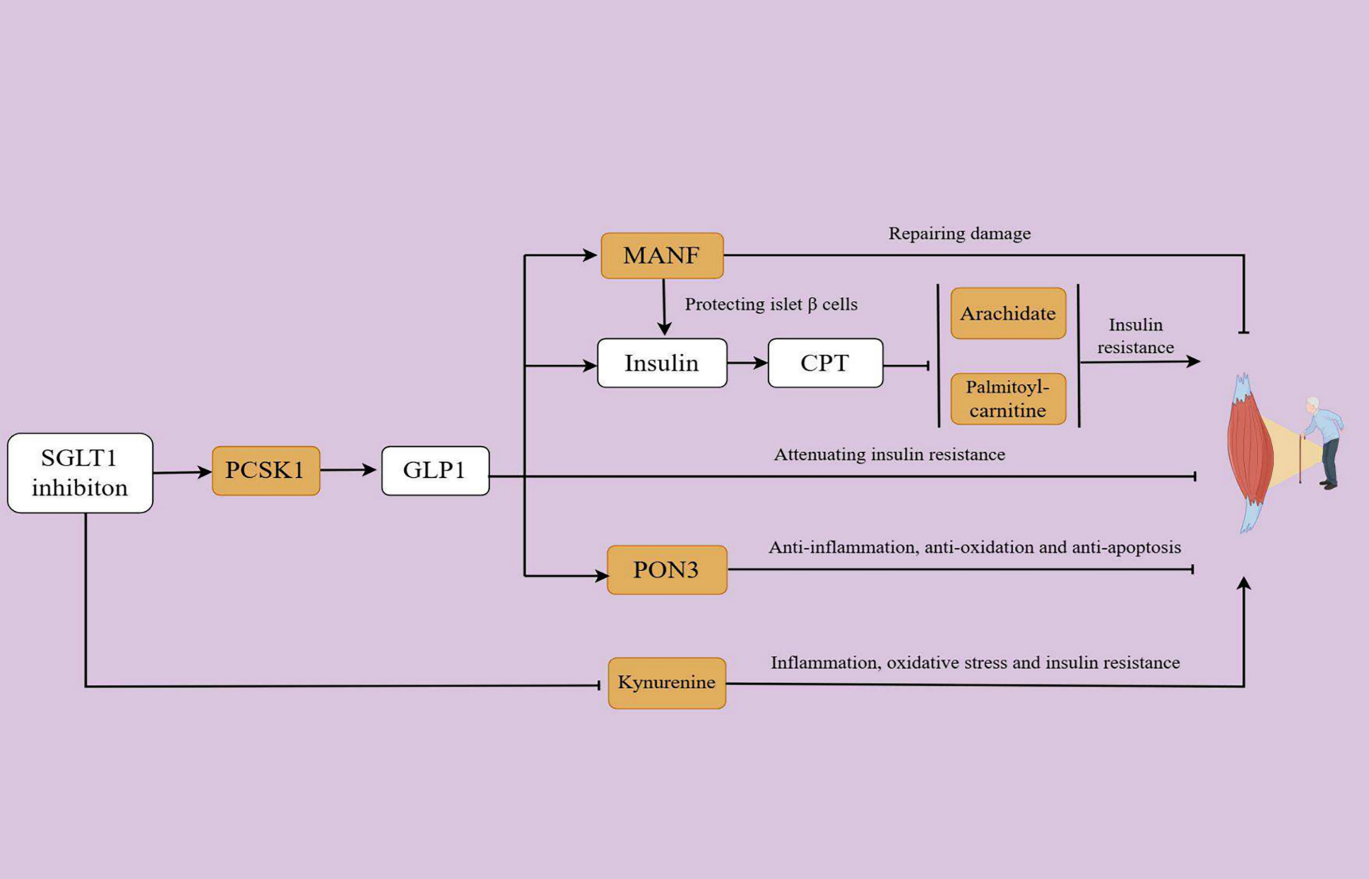
Roadmap suggesting potential mechanisms of SGLT1 inhibition on attenuating frailty through mediators. SGLT1 inhibition led to increased expression of PCSK1 which in turn up‐regulated the GLP1 levels. The interaction between GLP1 and its receptor in skeletal muscle could attenuate the insulin resistance. Moreover, GLP1 acted as a stimulus for insulin secretion which could activate the CPT expression, consequently inhibiting the insulin resistance in skeletal muscle induced by long chain saturated fatty acid, such as palmitoylcarnitine and arachidate. In addition, the activation of GLP1 increased the MANF level, facilitating the repair of skeletal muscle damage and promoting the survival of pancreatic β cells, which were responsible for insulin secretion. Meanwhile, GLP1 also increased the expression levels of PON3, which could protect the skeletal muscle by anti‐inflammation, anti‐oxidation and anti‐apoptosis. In addition, SGLT1 inhibition suppressed the level of kynurenine, a molecule participating in the biological process of inflammation, oxidative stress and insulin resistance, thereby safeguarding skeletal muscle health. CPT, carnitine palmitoyltransferase; GLP1, glucagon‐like peptide‐1; MANF, mesencephalic astrocyte‐derived neurotrophic factor; PCSK1, neuroendocrine convertase 1; PON3, serum paraoxonase/lactonase 3; SGLT1, sodium‐glucose co‐transporter 1.

After MR analysis, 167 proteins were found to be associated with FI, with 79 proteins showing positive associations and 88 proteins showing negative associations (Figure 3A, Table S10). Then we further estimated the effect of SGLT1 inhibition on these screened proteins and identified 24 proteins as mediators of the protecting effect of SGLT1 inhibition on frailty. For instance, neuroendocrine convertase 1 (PCSK1), mesencephalic astrocyte‐derived neurotrophic factor (MANF) and serum paraoxonase/lactonase 3 (PON3) were identified as mediators, accounting for 13.49% (95% CI: 5.08%, 21.91%), 18.94% (95% CI: 0.93%, 36.95%) and 44.90% (95% CI: 10.90%, 78.90%) of the total effect of SGLT1 inhibition on FI, respectively (Figure 4, Table S9).

**FIGURE 5:**
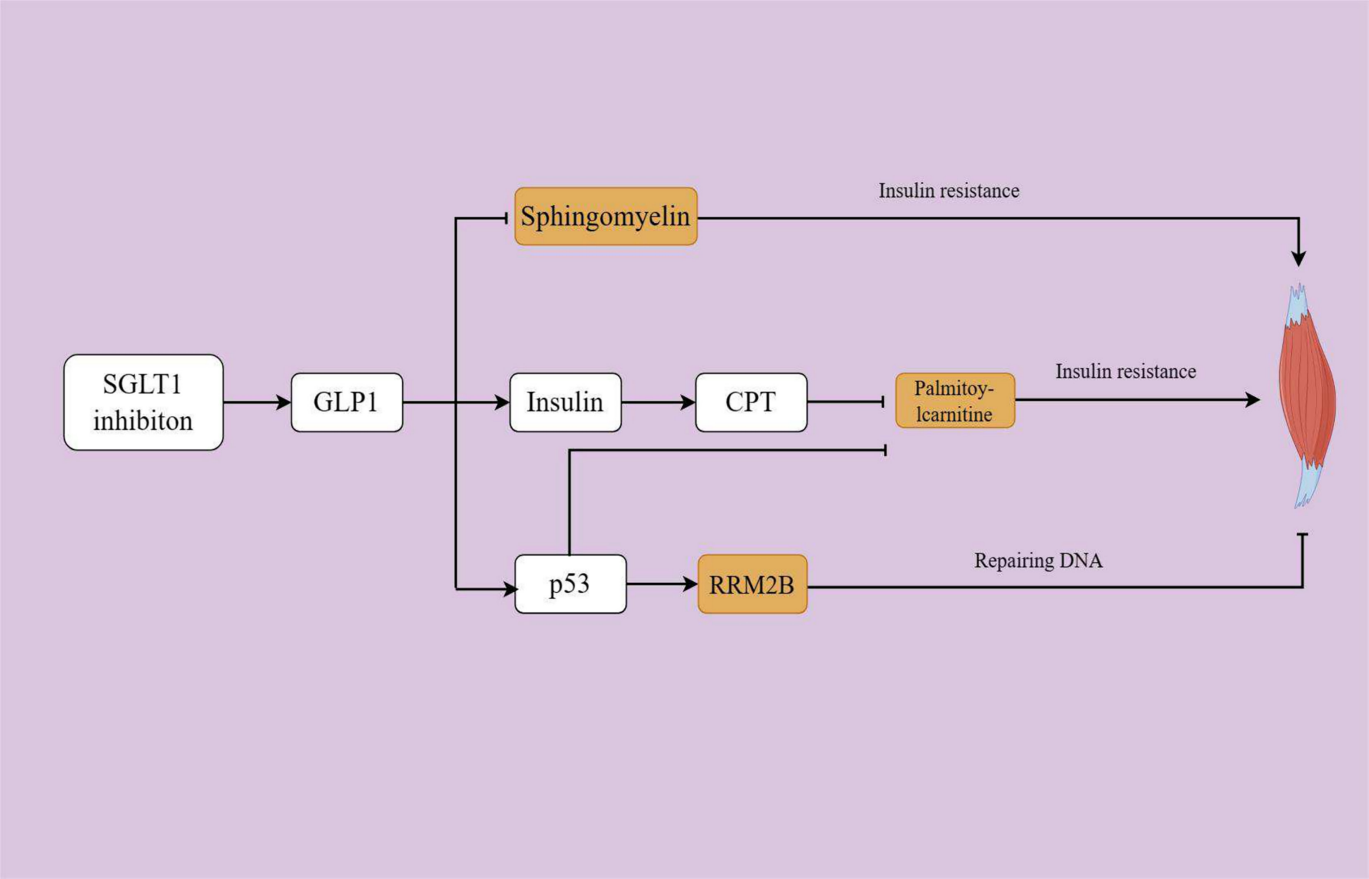
Roadmap suggesting potential mechanisms of SGLT1 inhibition on attenuating sarcopenia through mediators. GLP1 activation, promoted by SGLT1 inhibition, decreased the level of sphingomyelin that was widely recognized as a trigger of insulin resistance in skeletal muscle. In addition, the activation of GLP1 promoted the insulin secretion and CPT upregulation, thereby mitigating palmitoylcarnitine‐induced insulin resistance in skeletal muscle. Additionally, GLP1 was demonstrated to be related to higher level of p53 which in turn increased the RRM2B expression, facilitating the DNA repair of skeletal muscle. In particular, p53 also showed the ability of attenuating palmitoylcarnitine‐induced insulin resistance in skeletal muscle. CPT, carnitine palmitoyltransferase; DNA, deoxyribonucleic acid; GLP1, glucagon‐like peptide‐1; SGLT1, sodium‐glucose co‐transporter 1.

Through MR analysis, 143 proteins were found to be linked to low grip strength under the FNIH criteria, with 72 showing positive associations and 71 showing negative associations (Figure 3B, Table S11). Similarly, 163 proteins were demonstrated to be associated with low grip strength under the EWGSOP criteria, with 75 displaying positive associations and 88 displaying negative associations (Figure 3C, Table S12). Then we assessed the causal effect of SGLT1 inhibition on these screened proteins in order to identify potential mediators. Consequently, 13 proteins were identified as mediators of the effect of SGLT1 inhibition on low grip strength under the FNIH criteria while 22 proteins were identified as mediators of the effect of SGLT1 inhibition on low grip strength under the EWGSOP criteria. Specifically, ribonucleoside‐diphosphate reductase subunit M2 B (RRM2B) was found to mediate the effect of SGLT1 inhibition on low grip strength under both FNIH and EWGSOP criteria, explaining 26.98% (95% CI: 6.69%, 47.26%) and 44.06% (95% CI: 6.28%, 81.83%) of the overall effect, respectively (Figure 5, Table S9). Different GWAS databases of protein mediators were used in the validation analysis to strengthen the credibility of the mediating effects. The results validated the mediating effects of nine protein mediators on the relation between SGLT1 inhibition and outcomes (Table S13).

Further investigation of protein mediators involving in the inhibiting effect of SGLT1 on low grip strength across genders was performed. Eleven proteins were identified as mediators of the effect of SGLT1 inhibition on low grip strength in males while 16 proteins were proved to be mediators in females (Tables S9, S14–S15).

### 3.4 Mediation MR of SGLT1 Inhibition, Plasma Metabolites and FI as Well as Low Grip Strength

We subsequently examined the correlation between 1352 metabolites and outcomes, revealing that 113 metabolites were linked to FI, with 63 showing positive associations and 50 showing negative associations (Figure 3D, Table S16). Among these metabolites, 16 were identified as mediators of the effect of SGLT1 inhibition on FI. For instance, SGLT1 inhibition was related with decreased levels of kynurenine, palmitoylcarnitine and arachidate, all of which were strongly correlated with FI, explaining 12.11% (95% CI: 3.27%, 20.95%), 9.69% (95% CI: 1.28%, 18.11%) and 9.59% (95% CI: 0.23%, 18.94%) of the total effect, respectively (Figure 4, Table S9).

Similarly, metabolite–phenotype association was accessed to identify potential mediators of the impact of SGLT1 inhibition and low grip strength in individuals aged 60 years and older under both FNIH and EWGSOP criteria. After screening, it was found that 80 metabolites were significantly correlated with low grip strength according to the FNIH criteria, with 46 exhibiting positive associations and 34 exhibiting negative associations (Figure 3E, Table S17). Additionally, 70 metabolites were screened to be associated with low grip strength under EWGSOP criteria, with 34 showing positive associations and 36 showing negative associations (Figure 3F, Table S18). Then the causal effect of SGLT1 inhibition on the metabolites that significantly associated with outcomes was assessed to identify the potential mediators. As a result, we identified 16 metabolites mediating the effect of SGLT1 inhibition on low grip strength under FNIH criteria and 6 metabolites mediating the effect of SGLT1 inhibition on low grip strength under EWGSOP criteria. Among these metabolites, palmitoylcarnitine, which was the mediator of FI, also played a key role in mediating the impact of SGLT1 inhibition on low grip strength, contributing to 8.35% (95% CI: 0.21%, 16.48%) of the total effect. Additionally, metabolites involved in lipid metabolism, such as sphingomyelin, were also identified as contributors mediating the effect of SGLT1 inhibition on low grip strength (Figure 5, Table S9).

Specifically, gender differences in the mediating effects of metabolites were detected. In males, 10 metabolites were found to mediate the association between SGLT1 inhibition and low grip strength while in females, 12 metabolites were identified as mediators (Tables S9, S19–S20).

## 4 Discussion

In the present study, we found the protective role of SGLT1 inhibition on frailty and sarcopenia through MR analysis. Furthermore, we proved the intermediary role of insulin resistance on the association between SGLT1 inhibition and frailty, suggesting that the attenuation of frailty by SGLT1 inhibition was possibly mediated by the alleviation of insulin resistance. After screening, a series of proteins and metabolites were identified to mediate the effect of SGLT1 inhibition on frailty and sarcopenia, indicating plausible mechanistic pathways.

The effect of SGLT1 inhibition on the presence of frailty and sarcopenia was seldom discussed previously. It was well known that SGLT1, together with SGLT2, played an essential role in human physiological function by facilitating the cellular uptake of glucose alongside sodium ions [29]. SGLT2 inhibitor has been widely used in type 2 diabetes patients because of its ability to reduce glucose re‐absorption from urine [30]. However, recent studies indicated a better glucose‐lowering effect of dual inhibitor of both SGLT1 and SGLT2 [31]. Apart from the glucose‐lowering effect, the dual inhibitor was recently reported to attenuate the injuries of skeletal muscle caused by ischaemia in diabetic mice and decrease the impairment of C2C12 cells induced by high glucose and hypoxia [10]. This finding sheds light on the potential effect of SGLT1 and SGLT2 inhibition on protecting skeletal muscle cells, offering a novel approach to address ageing and degeneration in skeletal muscle, such as frailty and sarcopenia. Although existing researches have highlighted the protective role of SGLT2 inhibition on the development of frailty and sarcopenia, the influence of SGLT1 inhibition on these conditions has been relatively unexplored. In the present study, we explored the casual relationship between SGLT1 inhibition and FI as well as low grip strength using MR analysis and found out strong associations, indicating that SGLT1 inhibition could ameliorate frailty and sarcopenia.

In this study, we demonstrated the crucial role of insulin resistance in mediating the protective effect of SGLT1 inhibition on frailty. Insulin resistance, characterized by impaired insulin‐mediated regulation of glucose metabolism in tissues, was usually caused by disturbances in downstream signalling pathways of insulin [32]. Skeletal muscle was recognized as a targeted tissue for insulin simulation as well as a primary regulator of glycaemic control. It was well established that insulin played a crucial role in promoting glucose uptake and protein synthesis in skeletal muscle, thus contributing to the maintenance of physiological function of skeletal muscle [33]. Insulin sensitivity of the skeletal muscle declines with age, which was probably caused by mitochondrial dysfunction, β‐oxidation of fatty acids disorder, reactive oxygen species (ROS) production and inflammatory factors secretion, finally leading to skeletal muscle dysfunction and frailty [34, 35, 36, 37]. SGLT1 inhibition could increase insulin secretion to alleviate insulin resistance by several intracellular mechanisms. Upon exposure to high levels of glucose, SGLT1 in pancreatic α cells facilitated sodium influx, affecting the function of sodium proton exchangers and resulting in the decrease of cellular PH levels. Consequently, PH‐sensitive metabolic enzymes were activated, prompting the release of glucagon and disrupting the secretion of insulin, which was suppressed by SGLT1 inhibition [38, 39]. Additionally, inhibition of SGLT1 in the intestine was proved to promote the glucagon‐like peptide‐1 (GLP1) secretion, which in turn, stimulated the β cells to release insulin [40, 41]. Recently, Wu et al. have detected the expression of receptors of GLP1 in the skeletal muscle of mice. Their research indicated that over‐expression of GLP1 in the skeletal muscle improved the endurance capacity through regulating glycogen synthesis, enhancing glucose uptake, increasing mitochondrial biogenesis and elevating oxidative metabolism [42]. This finding laid the foundation that high levels of GLP1 resulting from SGLT1 inhibition improved insulin sensitivity and physiological function of skeletal muscle.

In this paper, we further confirmed that in the casual association between SGLT1 inhibition and FI, PON3 explained 44.90% of total casual effect, followed by MANF (18.94%), PCSK1 (13.49%), kynurenine (12.11%), palmitoylcarnitine (9.69%) and arachidate (9.59%). Specifically, PON3, as one of members of the PON family of hydrolytic enzymes, was a calcium‐dependent glycoprotein synthesized in the liver, playing a role of hydrolysing lactones and eicosanoids [43]. PON3 has been reported to exhibit the anti‐inflammatory [44], antioxidant [45] and anti‐apoptotic [46] abilities. Moreover, the expression of PON3 has been detected in skeletal muscle [47], and the upregulation of PON3 was demonstrated to promote the proliferation of bovine skeletal muscle satellite cells [48]. These findings probably explained the protective role of PON3 in skeletal muscle. Additionally, liraglutide, a GLP1 receptor agonist, has been reported to elevate the expression of PON3 [49], indicating that inhibiting SGLT1 possibly led to an increase of PON3 expression in the skeletal muscle through the elevation of GLP1 levels. Hence, PON3 may establish the connection between SGLT1 inhibition and skeletal muscle ageing.

Basically, MANF was a member of the new family of neurotrophic factors (NTFs) and broadly expressed in mature tissues [50]. A recent research has reported a notable increase in MANF levels after skeletal muscle injury, which facilitated the repair processes of skeletal muscle. However, in aged animals, the increase in MANF after injury was suppressed. This finding underscored the essential role of MANF in skeletal muscle injury repair [51]. Moreover, MANF has been shown to protect β cells from endoplasmic reticulum stress [52], potentially enhancing insulin sensitivity in skeletal muscle. Furthermore, the activation of the GLP1 receptor, a downstream signalling pathway of SGLT1 inhibition, was reported to trigger increased expression of MANF [52], which may partly explain the beneficial effect of SGLT1 inhibition on frailty.

Prohormone convertase 1/3 (PC1/3), encoded by the PCSK1, was a serine endoprotease [53], playing an important role in processing GLP1 [54]. Researches have indicated that the inhibition of SGLT2, a closely related member of the SGLT family, had the potential to enhance PCSK1 expression and subsequently elevated GLP1 levels [55]. Therefore, the inhibition of another member of SGLTs, SGLT1, may exhibit a similar effect. Additionally, PCSK1 was also proved to be expressed in the skeletal muscle (https://www.genecards.org/cgi‐bin/carddisp.pl?gene=PCSK1). On the basis of the evidences, it was suggested that SGLT1 inhibition probably elevated the GLP1 level through the upregulation of PCSK1 in the skeletal muscle, potentially contributing to alleviation in frailty.

Kynurenine, one of the metabolic mediators identified in our investigation, was generated by tryptophan metabolism [56]. Studies showed that kynurenine levels increased with age and led to muscle loss [57, 58]. Furthermore, Valdiglesias et al. reported a strong relationship between plasma kynurenine level and frailty in elderly people [59], which was consistent with the present study. Kynurenine increased the level of oxidative stress, inflammation and insulin resistance [57, 58, 60, 61, 62], elucidating its possible role in the impairment of skeletal muscle function. In addition, a recent study utilized muscle biopsies to examine metabolic characteristics in skeletal muscles of heart failure patients treated with or without SGLT2 inhibitors. The results indicated a significant reduction of kynurenine levels in skeletal muscles and alleviation of skeletal muscle atrophy, after SGLT2 inhibitor treatment [63]. It was well documented that SGLT2 inhibitors also exhibited partial inhibition effect on SGLT1 due to their pharmacological similarity [64]. Therefore, we hypothesized that SGLT1 inhibition suppressed kynurenine level and eliminated its deleterious effect on skeletal muscle.

As was shown, lipid metabolism played an important role in mediating the effect of SGLT1 inhibition on frailty. Emerging evidence suggested that fatty acids were significantly related with frailty in elderly [65]. Our data also demonstrated that palmitoylcarnitine and arachidate, recognized as metabolic products of long‐chain saturated fatty acids, was positively associated with frailty. In essence, long‐chain saturated fatty acids have been shown to induce insulin resistance in skeletal muscle [66, 67, 68]. Fatty acids and their metabolites were transported into the mitochondria for β‐oxidation, which was facilitated by carnitine palmitoyltransferase (CPT) [69, 70]. Moreover, CPT was crucial in safeguarding muscle tissue against insulin resistance by promoting the metabolism of long‐chain saturated fatty acids [71, 72]. Additionally, insulin signals upregulated the level of CPT, promoting the β‐oxidation of fatty acid in skeletal muscle [73]. It was possible that inhibiting SGLT1 may have reduced the levels of palmitoylcarnitine and arachidate in skeletal muscle by stimulating insulin secretion and activating CPT, ultimately mitigating frailty.

Our study identified several proteins and metabolites, such as RRM2B, palmitoylcarnitine and sphingomyelin, that were found to be associated with low grip strength. Of particular interest was the observation that palmitoylcarnitine was related with both FI and low grip strength, indicating the crucial role of long‐chain saturated fatty acid in aged‐related muscle dysfunction. Actually, the accumulation of lipid in skeletal muscle was reported to promote the muscle atrophy by inducing insulin resistance [74]. Our data further supported the previous findings, suggesting that palmitoylcarnitine served as a mediator in the impact of SGLT1 inhibition on both frailty and sarcopenia.

Additionally, sphingomyelin, a key structural component of biological membranes, was found to be positively correlated with sarcopenia in the current investigation. It has been well established that sphingomyelin and its metabolic derivatives acted as secondary messengers in multiple tissues, playing a role in the development of insulin resistance [74]. In particular, the sphingomyelin signalling pathway in muscle has been identified as a determinant of insulin resistance in humans [75]. Inhibition of the acid sphingomyelinase was reported to improve the insulin sensitivity in elderly individuals [76]. In our study, we demonstrated that sphingomyelin played an intermediary role in the relationship between SGLT1 inhibition and low grip strength. GLP1 receptor activation could reduce the accumulation of sphingomyelin and its metabolic products [77]. Therefore, SGLT1 inhibition may suppress the sphingomyelin level by elevating the level of GLP1, subsequently improved the grip strength.

RRM2B was recognized as one of the mediators participating in the effect of SGLT1 inhibition on sarcopenia. It was a critical ribonucleotide reductase subunit, playing an essential role in DNA synthesis and repair in p53‐dependent manner [78, 79]. Researches have demonstrated that patients with mutations in RRM2B experienced impaired mitochondrial DNA synthesis in skeletal muscle, resulting in serious dysfunction of skeletal muscle [80, 81]. Furthermore, the knockout of RRM2B in myofibres resulted in weakness of muscles and triggered the differentiation of skeletal muscle stem cells (MuSCs) [82]. p53, widely acknowledged as a classical biomarker of ageing, exhibited the capacity to impede cell proliferation in response to DNA damage and initiated DNA repair by activating RRM2B, thereby promoting cell survival [83, 84, 85]. It was suggested that the p53/RRM2B pathway played a critical role in the survival of skeletal muscle during ageing. Moreover, p53 was reported to attenuate the insulin resistance induced by palmitate through NF‐κB and p38/ERK MAPK pathways [86]. These findings provided a reliable evidence that p53 exhibited a beneficial effect on skeletal muscle by either promoting DNA repair through activation of RRM2B or ameliorating insulin resistance, which in turn mitigated the sarcopenia.

The crosstalk between frailty and sarcopenia has been extensively studied and confirmed in our research. For example, asparagine was identified as an amino acid‐related metabolite negatively correlated with both frailty and sarcopenia in our study. Previous studies revealed a negative correlation between asparagine levels and frailty in the elderly [87] and a decrease of asparagine levels in skeletal muscle of elderly individuals with sarcopenia [88]. Moreover, supplementation of asparagine in the diet enhanced the capacity of the muscle to utilize free fatty acids and spare glycogen, prolonging the exhaustion time during exercise [89], indicating a crucial role of asparagine in both frailty and sarcopenia. In our study, the effects of SGLT1 inhibition on both frailty and sarcopenia shared some co‐mediators. Specifically, palmitoylcarnitine, stearoylcarnitine, ximenoylcarnitine and 3‐hydroxy‐3‐methylglutarate, all identified as lipid metabolites, mediated the impact of SGLT1 inhibition on both conditions. It may be attributed to the fact that SGLT1 inhibitor suppressed the lipid‐induced insulin resistance. In addition, our study also found that some metabolic byproducts of plasma membrane played a crucial role in mediating the SGLT1 inhibition's effect on both frailty and sarcopenia. For frailty, phosphatidylcholine (GPC), phosphatidylethanolamine (GPE) and phosphatidylinositol (GPI) were demonstrated to be mediators. Consistently, previous studies have shown that these byproducts of phospholipids exhibited an inverse correlation with muscle volume and peak power, contributing to the decline in skeletal muscle mass and function among elderly individuals [90]. However, sphingomyelin, also known as a component of plasma membrane, was identified as a key mediator for low grip strength which participated in insulin resistance. Some proteins with the ability of repairing injury and maintaining survival of skeletal muscle also played a role. Specifically, MANF was demonstrated as a mediator of FI, while RRM2B mediated the effect of SGLT1 inhibition on low grip strength. Molecules involved in biological process of inflammation, oxidative stress and apoptosis, like PON3 and kynurenine, were demonstrated as mediators of FI rather than low grip strength. It may be because low grip strength, which was recognized as an important indicator of early stage of ageing [91], has not yet developed many detectable changes, such as inflammation, oxidative stress and apoptosis [92].

We further analysed the potential proteins and metabolites that were associated with low grip strength in both genders. Specifically, SERPING1 showed a close association with low grip strength in individuals of both genders. It was known for the capacity of regulation of vascular permeability and suppression of inflammation [93], probably exhibiting some beneficial effects on skeletal muscle. Studies have indicated that SERPING1 mitigated the reperfusion injury of skeletal muscle in rats [94], which may explain its role of alleviating sarcopenia. SERPING1 was also identified as mediators of the effects of SGLT1 inhibition on low grip strength across genders, indicating that SGLT1 inhibition attenuated the low grip strength in different genders through the common targets.

Our research acknowledged several limitations. First, the potential effects of protein levels within specific cells and tissues have not been adequately investigated because the abundances of these circulating proteins may differ from those within cells and tissues. Second, despite employing multiple MR approaches to address pleiotropy confounding, residual bias persisted. Third, although sensitivity and validation analyses were conducted to ensure the reliability of our findings, further experiments in vivo and vitro were needed to be explored.

## 5 Conclusion

In conclusion, SGLT1 inhibition exhibited a protective role in attenuating frailty and sarcopenia. Furthermore, potential mediators of these effects were involved in various biological processes.

## Conflicts of Interest

The authors declare no conflicts of interest.

## Supporting information

Supporting Information.

**Table S1.** Detailed information for genome‐wide association study (GWAS) statistics used in the present study.
**Table S2.** Instrumental variables for SGLT1 inhibition in primary analysis.
**Table S3.** Instrumental variables for metabolites.
**Table S4.** Questionnaire items from UK Biobank and TwinGene used to compose the FI.
**Table S5.** Instrumental variables for SGLT1 inhibition in validation analysis.
**Table S6.** Directional pleiotropy test and heterogeneity test for the causal association between SGLT1 inhibition and FI / low hand grip strength in primary analysis.
**Table S7.** Heterogeneity test for the causal association between SGLT1 inhibition and FI / low hand grip strength in validation analysis.
**Table S8.** MR estimates of the insulin resistance phenotype on FI.
**Table S9.** Proportion of mediators in the causal associations between SGLT1 inhibiton and FI / low hand grip strength.
**Table S10.** MR estimates of the proteins on FI.
**Table S11.** MR estimates of the proteins on low hand grip strength‐total (60 years and older) (FNIH).
**Table S12.** MR estimates of the proteins on low hand grip strength‐total (60 years and older) (EWGSOP).
**Table S13.** Proportion of validated mediators in the causal associations between SGLT1 inhibiton and FI / low hand grip strength from different databases.
**Table S14.** MR estimates of the proteins on low hand grip strength‐male (60 years and older) (FNIH).
**Table S15.** MR estimates of the proteins on low hand grip strength‐female (60 years and older) (FNIH).
**Table S16.** MR estimates of the metabolites on FI.
**Table S17.** MR estimates of the metabolites on low hand grip strength‐total (60 years and older) (FNIH).
**Table S18.** MR estimates of the metabolites on low hand grip strength‐total (60 years and older) (EWGSOP).
**Table S19.** MR estimates of the metabolites on low hand grip strength‐male (60 years and older) (FNIH).
**Table S20.** MR estimates of the metabolites on low hand grip strength‐female (60 years and older) (FNIH).

## Data Availability

The GWAS Summary statistics used in this study were publicly accessed from the IEU OpenGWAS project (https://gwas.mrcieu.ac.uk/), GWAS Catalog (https://www.ebi.ac.uk/gwas/), deCODE genetics (https://www.decode.com/summarydata/) and LBC 1936 (https://datashare.ed.ac.uk/handle/10283/3408).

## References

[1] L. C. Behr , A. Simm , A. Kluttig , and A. Grosskopf , “60 Years of Healthy Aging: On Definitions, Biomarkers, Scores and Challenges,” Ageing Research Reviews 88 (2023): 101934. 37059401 10.1016/j.arr.2023.101934

[2] R. M. Collard , H. Boter , R. A. Schoevers , and R. C. Oude Voshaar , “Prevalence of Frailty in Community‐Dwelling Older Persons: A Systematic Review,” Journal of the American Geriatrics Society 60 (2012): 1487–1492. 22881367 10.1111/j.1532-5415.2012.04054.x

[3] A. J. Mayhew , K. Amog , S. Phillips , et al., “The Prevalence of Sarcopenia in Community‐Dwelling Older Adults, an Exploration of Differences Between Studies and Within Definitions: A Systematic Review and Meta‐Analyses,” Age and Ageing 48 (2019): 48–56. 30052707 10.1093/ageing/afy106

[4] E. Carmeli , “Frailty and Primary Sarcopenia: A Review,” Advances in Experimental Medicine and Biology 1020 (2017): 53–68. 28382607 10.1007/5584_2017_18

[5] A. Álvarez‐Bustos , J. A. Carnicero‐Carreño , B. Davies , et al., “Role of Sarcopenia in the Frailty Transitions in Older Adults: A Population‐Based Cohort Study,” Journal of Cachexia, Sarcopenia and Muscle 13 (2022): 2352–2360. 35903871 10.1002/jcsm.13055 PMC9530539

[6] L. Ye , R. Liang , X. Liu , J. Li , J. Yue , and X. Zhang , “Frailty and Sarcopenia: A Bibliometric Analysis of Their Association and Potential Targets for Intervention,” Ageing Research Reviews 92 (2023): 102111. 38031836 10.1016/j.arr.2023.102111

[7] A. Picca , H. J. Coelho‐Junior , R. Calvani , E. Marzetti , and D. L. Vetrano , “Biomarkers Shared by Frailty and Sarcopenia in Older Adults: A Systematic Review and Meta‐Analysis,” Ageing Research Reviews 73 (2022): 101530. 34839041 10.1016/j.arr.2021.101530

[8] D. Wilson , T. Jackson , E. Sapey , and J. M. Lord , “Frailty and Sarcopenia: The Potential Role of an Aged Immune System,” Ageing Research Reviews 36 (2017): 1–10. 28223244 10.1016/j.arr.2017.01.006

[9] E. Massimino , A. Izzo , G. Riccardi , and P. G. Della , “The Impact of Glucose‐Lowering Drugs on Sarcopenia in Type 2 Diabetes: Current Evidence and Underlying Mechanisms,” Cells 10 (2021): 1958. 34440727 10.3390/cells10081958 PMC8393336

[10] L. L. Luo , J. X. Han , S. R. Wu , and V. Kasim , “Intramuscular Injection of Sotagliflozin Promotes Neovascularization in Diabetic Mice Through Enhancing Skeletal Muscle Cells Paracrine Function,” Acta Pharmacologica Sinica 43 (2022): 2636–2650. 35292769 10.1038/s41401-022-00889-4 PMC9525294

[11] A. Kutz , D. H. Kim , D. J. Wexler , et al., “Comparative Cardiovascular Effectiveness and Safety of SGLT‐2 Inhibitors, GLP‐1 Receptor Agonists, and DPP‐4 Inhibitors According to Frailty in Type 2 Diabetes,” Diabetes Care 46 (2023): 2004–2014. 37677118 10.2337/dc23-0671 PMC10620535

[12] S. J. Wood , J. S. Bell , D. J. Magliano , J. E. Shaw , M. Cesari , and J. Ilomaki , “Effectiveness of Sodium‐Glucose Cotransporter‐2 Inhibitors vs. Dipeptidyl Peptidase‐4 Inhibitors in Frail People With Diabetes Who Were Recently Hospitalized,” Frontiers in Pharmacology 13 (2022): 886834. 35903329 10.3389/fphar.2022.886834 PMC9315378

[13] M. Sano , S. Meguro , T. Kawai , and Y. Suzuki , “Increased Grip Strength With Sodium‐Glucose Cotransporter 2,” Journal of Diabetes 8 (2016): 736–737. 27038414 10.1111/1753-0407.12402

[14] T. Okamura , Y. Hashimoto , T. Osaka , T. Fukuda , M. Hamaguchi , and M. Fukui , “The Sodium‐Glucose Cotransporter 2 Inhibitor Luseogliflozin Can Suppress Muscle Atrophy in Db/Db Mice by Suppressing the Expression of *foxo1* ,” Journal of Clinical Biochemistry and Nutrition 65 (2019): 23–28. 31379410 10.3164/jcbn.18-114 PMC6667382

[15] D. P. MacKinnon , C. M. Lockwood , J. M. Hoffman , S. G. West , and V. Sheets , “A Comparison of Methods to Test Mediation and Other Intervening Variable Effects,” Psychological Methods 7 (2002): 83–104. 11928892 10.1037/1082-989x.7.1.83 PMC2819363

[16] GTEx Consortium , “The GTEx Consortium Atlas of Genetic Regulatory Effects Across Human Tissues,” Science 369 (2020): 1318–1330. 32913098 10.1126/science.aaz1776 PMC7737656

[17] T. Rieg and V. Vallon , “Development of SGLT1 and SGLT2 Inhibitors,” Diabetologia 61 (2018): 2079–2086. 30132033 10.1007/s00125-018-4654-7 PMC6124499

[18] S. S. Zhao , S. Rajasundaram , V. Karhunen , U. Alam , and D. Gill , “Sodium‐Glucose Cotransporter 1 Inhibition and Gout: Mendelian Randomisation Study,” Seminars in Arthritis and Rheumatism 56 (2022): 152058. 35839537 10.1016/j.semarthrit.2022.152058

[19] M. Xu , J. Zheng , T. Hou , et al., “SGLT2 Inhibition, Choline Metabolites, and Cardiometabolic Diseases: A Mediation Mendelian Randomization Study,” Diabetes Care 45 (2022): 2718–2728. 36161993 10.2337/dc22-0323 PMC9862376

[20] T. M. Palmer , D. A. Lawlor , R. M. Harbord , et al., “Using Multiple Genetic Variants as Instrumental Variables for Modifiable Risk Factors,” Statistical Methods in Medical Research 21 (2012): 223–242. 21216802 10.1177/0962280210394459 PMC3917707

[21] E. Dent , P. Kowal , and E. O. Hoogendijk , “Frailty Measurement in Research and Clinical Practice: A Review,” European Journal of Internal Medicine 31 (2016): 3–10. 27039014 10.1016/j.ejim.2016.03.007

[22] J. L. Atkins , J. Jylhävä , N. L. Pedersen , et al., “A Genome‐Wide Association Study of the Frailty Index Highlights Brain Pathways in Ageing,” Aging Cell 20 (2021): e13459. 34431594 10.1111/acel.13459 PMC8441299

[23] R. R. McLean , M. D. Shardell , D. E. Alley , et al., “Criteria for Clinically Relevant Weakness and Low Lean Mass and Their Longitudinal Association With Incident Mobility Impairment and Mortality: The Foundation for the National Institutes of Health (FNIH) Sarcopenia Project,” Journals of Gerontology. Series a, Biological Sciences and Medical Sciences 69 (2014): 576–583. 24737560 10.1093/gerona/glu012 PMC3991140

[24] A. J. Cruz‐Jentoft , J. P. Baeyens , J. M. Bauer , et al., “Sarcopenia: European Consensus on Definition and Diagnosis: Report of the European Working Group on Sarcopenia in Older People,” Age and Ageing 39 (2010): 412–423. 20392703 10.1093/ageing/afq034 PMC2886201

[25] G. Jones , K. Trajanoska , A. J. Santanasto , et al., “Genome‐Wide Meta‐Analysis of Muscle Weakness Identifies 15 Susceptibility Loci in Older Men and Women,” Nature Communications 12 (2021): 654. 10.1038/s41467-021-20918-w PMC7844411 33510174

[26] Z. Lin , Y. Deng , and W. Pan , “Combining the Strengths of Inverse‐Variance Weighting and Egger Regression in Mendelian Randomization Using a Mixture of Regressions Model,” PLoS Genetics 17 (2021): e1009922. 34793444 10.1371/journal.pgen.1009922 PMC8639093

[27] M. Verbanck , C. Y. Chen , B. Neale , and R. Do , “Detection of Widespread Horizontal Pleiotropy in Causal Relationships Inferred From Mendelian Randomization Between Complex Traits and Diseases,” Nature Genetics 50 (2018): 693–698. 29686387 10.1038/s41588-018-0099-7 PMC6083837

[28] S. Burgess , G. Davey Smith , N. M. Davies , et al., “Guidelines for Performing Mendelian Randomization Investigations: Update for Summer 2023,” Wellcome Open Research 4 (2019): 186. 32760811 10.12688/wellcomeopenres.15555.1 PMC7384151

[29] E. M. Wright , D. D. Loo , and B. A. Hirayama , “Biology of Human Sodium Glucose Transporters,” Physiological Reviews 91 (2011): 733–794. 21527736 10.1152/physrev.00055.2009

[30] E. Ferrannini , “Sodium‐Glucose Co‐Transporters and Their Inhibition: Clinical Physiology,” Cell Metabolism 26 (2017): 27–38. 28506519 10.1016/j.cmet.2017.04.011

[31] B. Pitt and D. L. Bhatt , “Does SGLT1 Inhibition Add Benefit to SGLT2 Inhibition in Type 2 Diabetes?,” Circulation 144 (2021): 4–6. 33887961 10.1161/CIRCULATIONAHA.121.054442

[32] D. E. James , J. Stöckli , and M. J. Birnbaum , “The Aetiology and Molecular Landscape of Insulin Resistance,” Nature Reviews. Molecular Cell Biology 22 (2021): 751–771. 34285405 10.1038/s41580-021-00390-6

[33] L. Sylow , V. L. Tokarz , E. A. Richter , and A. Klip , “The Many Actions of Insulin in Skeletal Muscle, the Paramount Tissue Determining Glycemia,” Cell Metabolism 33 (2021): 758–780. 33826918 10.1016/j.cmet.2021.03.020

[34] C. W. Li , K. Yu , N. Shyh‐Chang , et al., “Pathogenesis of Sarcopenia and the Relationship With Fat Mass: Descriptive Review,” Journal of Cachexia, Sarcopenia and Muscle 13 (2022): 781–794. 35106971 10.1002/jcsm.12901 PMC8977978

[35] J. Angulo , M. El Assar , A. Álvarez‐Bustos , and L. Rodríguez‐Mañas , “Physical Activity and Exercise: Strategies to Manage Frailty,” Redox Biology 35 (2020): 101513. 32234291 10.1016/j.redox.2020.101513 PMC7284931

[36] L. R. Perazza , H. M. Brown‐Borg , and L. V. Thompson , “Physiological Systems in Promoting Frailty,” Comprehensive Physiology 12 (2022): 3575–3620. 35578945 10.1002/cphy.c210034 PMC9531553

[37] A. Clegg and Z. Hassan‐Smith , “Frailty and the Endocrine System,” Lancet Diabetes and Endocrinology 6 (2018): 743–752. 30017798 10.1016/S2213-8587(18)30110-4

[38] J. G. Knudsen , A. Hamilton , R. Ramracheya , et al., “Dysregulation of Glucagon Secretion by Hyperglycemia‐Induced Sodium‐Dependent Reduction of ATP Production,” Cell Metabolism 29 (2019): 430–442.e4. 30415925 10.1016/j.cmet.2018.10.003 PMC6370947

[39] T. Suga , O. Kikuchi , M. Kobayashi , et al., “SGLT1 in Pancreatic α Cells Regulates Glucagon Secretion in Mice, Possibly Explaining the Distinct Effects of SGLT2 Inhibitors on Plasma Glucagon Levels,” Molecular Metabolism 19 (2019): 1–12. 30416006 10.1016/j.molmet.2018.10.009 PMC6323192

[40] A. Lehmann and P. J. Hornby , “Intestinal SGLT1 in Metabolic Health and Disease,” American Journal of Physiology. Gastrointestinal and Liver Physiology 310 (2016): G887–G898. 27012770 10.1152/ajpgi.00068.2016

[41] R. Pais , F. M. Gribble , and F. Reimann , “Stimulation of Incretin Secreting Cells,” Therapeutic Advances in Endocrinology and Metabolism 7 (2016): 24–42. 26885360 10.1177/2042018815618177 PMC4740941

[42] L. Wu , M. Zhou , T. Li , et al., “GLP‐1 Regulates Exercise Endurance and Skeletal Muscle Remodeling via GLP‐1R/AMPK Pathway,” Biochimica et Biophysica Acta, Molecular Cell Research 1869 (2022): 119300. 35636559 10.1016/j.bbamcr.2022.119300

[43] C. J. Mohammed , S. Lamichhane , J. A. Connolly , et al., “A PON for All Seasons: Comparing Paraoxonase Enzyme Substrates, Activity and Action Including the Role of PON3 in Health and Disease,” Antioxidants (Basel) 11 (2022): 590. 35326240 10.3390/antiox11030590 PMC8945423

[44] D. M. Shih , J. M. Yu , L. Vergnes , et al., “PON3 Knockout Mice are Susceptible To Obesity, Gallstone Formation, and Atherosclerosis,” FASEB Journal 29 (2015): 1185–1197. 25477283 10.1096/fj.14-260570 PMC4396607

[45] J. G. Salazar , J. Marsillach , I. Reverte , et al., “Paraoxonase‐1 and ‐3 Protein Expression in the Brain of the Tg2576 Mouse Model of Alzheimer's Disease,” Antioxidants (Basel) 10 (2021): 339. 33668379 10.3390/antiox10030339 PMC7996151

[46] W. Peng , C. Zhang , H. Lv , et al., “Comparative Evaluation of the Protective Potentials Of Human Paraoxonase 1 and 3 Against CCl4‐Induced Liver Injury,” Toxicology Letters 193 (2010): 159–166. 20079818 10.1016/j.toxlet.2010.01.003

[47] J. Marsillach , B. Mackness , M. Mackness , et al., “Immunohistochemical Analysis Of Paraoxonases‐1, 2, and 3 Expression in Normal Mouse Tissues,” Free Radical Biology & Medicine 45 (2008): 146–157. 18440321 10.1016/j.freeradbiomed.2008.03.023

[48] T. Wang , Q. Niu , T. Zhang , et al., “Cis‐eQTL Analysis and Functional Validation of Candidate Genes for Carcass Yield Traits in Beef Cattle,” International Journal of Molecular Sciences 23 (2022): 15055. 36499383 10.3390/ijms232315055 PMC9736101

[49] Y. Liu , D. Zhu , G. Dong , Y. Zeng , P. Jiang , and Y. Xiao , “Liver Paraoxonase 3 Expression and the Effect of Liraglutide Treatment in a Rat Model of Diabetes,” Advances in Clinical and Experimental Medicine 30 (2021): 157–163. 33650330 10.17219/acem/130605

[50] B. Sivakumar and A. Krishnan , “Mesencephalic Astrocyte‐Derived Neurotrophic Factor (MANF): An Emerging Therapeutic Target for Neurodegenerative Disorders,” Cells 12 (2023): 1032. 37048105 10.3390/cells12071032 PMC10093115

[51] N. S. Sousa , M. F. Brás , I. B. Antunes , P. Lindholm , J. Neves , and P. Sousa‐Victor , “Aging Disrupts MANF‐Mediated Immune Modulation During Skeletal Muscle Regeneration,” Nature Aging 3 (2023): 585–599. 37118549 10.1038/s43587-023-00382-5

[52] J. Fu , K. M. Nchambi , H. Wu , X. Luo , X. An , and D. Liu , “Liraglutide Protects Pancreatic β Cells from Endoplasmic Reticulum Stress by Upregulating MANF to Promote Autophagy Turnover,” Life Sciences 252 (2020): 117648. 32275937 10.1016/j.lfs.2020.117648

[53] P. Stijnen , B. Ramos‐Molina , S. O'Rahilly , and J. W. Creemers , “PCSK1 Mutations and Human Endocrinopathies: From Obesity to Gastrointestinal Disorders,” Endocrine Reviews 37 (2016): 347–371. 27187081 10.1210/er.2015-1117

[54] A. Ramzy and T. J. Kieffer , “Altered Islet Prohormone Processing: A Cause or Consequence of Diabetes?” Physiological Reviews 102 (2022): 155–208. 34280055 10.1152/physrev.00008.2021

[55] R. Wei , X. Cui , J. Feng , et al., “Dapagliflozin Promotes Beta Cell Regeneration by Inducing Pancreatic Endocrine Cell Phenotype Conversion in Type 2 Diabetic Mice,” Metabolism 111 (2020): 154324. 32712220 10.1016/j.metabol.2020.154324

[56] T. W. Stone and R. O. Williams , “Modulation of T Cells by Tryptophan Metabolites in the Kynurenine Pathway,” Trends in Pharmacological Sciences 44 (2023): 442–456. 37248103 10.1016/j.tips.2023.04.006

[57] H. Kaiser , K. Yu , C. Pandya , et al., “Kynurenine, a Tryptophan Metabolite That Increases With Age, Induces Muscle Atrophy and Lipid Peroxidation,” Oxidative Medicine and Cellular Longevity 2019 (2019): 9894238. 31737181 10.1155/2019/9894238 PMC6815546

[58] J. M. Hinkley , G. X. Yu , R. A. Standley , et al., “Exercise and Ageing Impact the Kynurenine/Tryptophan Pathway and Acylcarnitine Metabolite Pools in Skeletal Muscle Of Older Adults,” Journal of Physiology 601 (2023): 2165–2188. 36814134 10.1113/JP284142 PMC10278663

[59] V. Valdiglesias , D. Marcos‐Pérez , M. Lorenzi , et al., “Immunological Alterations in Frail Older Adults: A Cross Sectional Study,” Experimental Gerontology 112 (2018): 119–126. 30240849 10.1016/j.exger.2018.09.010

[60] J. Ballesteros , D. Rivas , and G. Duque , “The Role of the Kynurenine Pathway in the Pathophysiology of Frailty, Sarcopenia, and Osteoporosis,” Nutrients 15 (2023): 3132. 37513550 10.3390/nu15143132 PMC10383689

[61] M. Priyadarshini , G. Navarro , D. J. Reiman , et al., “Gestational Insulin Resistance is Mediated by the Gut Microbiome‐Indoleamine 2,3‐Dioxygenase Axis,” Gastroenterology 162 (2022): 1675–1689.e11. 35032499 10.1053/j.gastro.2022.01.008 PMC9040389

[62] T. Huang , J. Song , J. Gao , et al., “Adipocyte‐Derived Kynurenine Promotes Obesity and Insulin Resistance by Activating the AhR/STAT3/IL‐6 Signaling,” Nature Communications 13 (2022): 3489. 10.1038/s41467-022-31126-5 PMC9205899 35715443

[63] N. Wood , S. Straw , C. W. Cheng , et al., “Sodium‐Glucose Cotransporter 2 Inhibitors Influence Skeletal Muscle Pathology in Patients with Heart Failure and Reduced Ejection Fraction,” European Journal of Heart Failure 26 (2024): 925–935. 38468429 10.1002/ejhf.3192

[64] X. Chen , X. Yu , G. Lian , et al., “Canagliflozin Inhibits PASMCs Proliferation Via Regulating SGLT1/AMPK Signaling and Attenuates Artery Remodeling in MCT‐Induced Pulmonary Arterial Hypertension,” Biomedicine & Pharmacotherapy 174 (2024): 116505. 38574614 10.1016/j.biopha.2024.116505

[65] P. Ahiawodzi , L. Djousse , J. H. Ix , et al., “Non‐Esterified Fatty Acids and Risks of Frailty, Disability, and Mobility Limitation in Older Adults: The Cardiovascular Health Study,” Journal of the American Geriatrics Society 68 (2020): 2890–2897. 32964434 10.1111/jgs.16793 PMC8285064

[66] J. A. Chavez and S. A. Summers , “Characterizing the Effects of Saturated Fatty Acids on Insulin Signaling and Ceramide and Diacylglycerol Accumulation in 3T3‐L1 Adipocytes and C2C12 Myotubes,” Archives of Biochemistry and Biophysics 419 (2003): 101–109. 14592453 10.1016/j.abb.2003.08.020

[67] A. Guerrero‐Hernández , D. Leon‐Aparicio , J. Chavez‐Reyes , J. A. Olivares‐Reyes , and S. DeJesus , “Endoplasmic Reticulum Stress in Insulin Resistance and Diabetes,” Cell Calcium 56 (2014): 311–322. 25239386 10.1016/j.ceca.2014.08.006

[68] E. Liepinsh , M. Makrecka‐Kuka , E. Makarova , et al., “Acute and Long‐Term Administration of Palmitoylcarnitine Induces Muscle‐Specific Insulin Resistance in Mice,” BioFactors 43 (2017): 718–730. 28759135 10.1002/biof.1378

[69] P. R. Joshi and S. Zierz , “Muscle Carnitine Palmitoyltransferase II (CPT II) Deficiency: A Conceptual Approach,” Molecules 25 (2020): 1784. 32295037 10.3390/molecules25081784 PMC7221885

[70] W. Zhuang , G. Lian , B. Huang , et al., “CPT1 Regulates the Proliferation of Pulmonary Artery Smooth Muscle Cells Through the AMPK‐p53‐p21 Pathway in Pulmonary Arterial Hypertension,” Molecular and Cellular Biochemistry 455 (2019): 169–183. 30511343 10.1007/s11010-018-3480-z

[71] M. L. Blackburn , K. D. Ono‐Moore , H. F. Sobhi , and S. H. Adams , “Carnitine Palmitoyltransferase 2 Knockout Potentiates Palmitate‐Induced Insulin Resistance in C(2)C(12) Myotubes,” American Journal of Physiology. Endocrinology and Metabolism 319 (2020): e265–e275. 32459525 10.1152/ajpendo.00515.2019

[72] D. Sebastián , L. Herrero , D. Serra , G. Asins , and F. G. Hegardt , “CPT I Overexpression Protects L6E9 Muscle Cells from Fatty Acid‐Induced Insulin Resistance,” American Journal of Physiology. Endocrinology and Metabolism 292 (2007): E677–E686. 17062841 10.1152/ajpendo.00360.2006

[73] P. M. Miotto , H. L. Petrick , and G. P. Holloway , “Acute Insulin Deprivation Results in Altered Mitochondrial Substrate Sensitivity Conducive to Greater Fatty Acid Transport,” American Journal of Physiology. Endocrinology and Metabolism 319 (2020): e345–e353. 32543943 10.1152/ajpendo.00495.2019 PMC7473910

[74] Y. Huang , T. Huang , X. Zhen , et al., “A selective Sphingomyelin Synthase 2 Inhibitor Ameliorates Diet Induced Insulin Resistance Via the IRS‐1/Akt/GSK‐3β Signaling Pathway,” Pharmazie 74 (2019): 553–558. 31484596 10.1691/ph.2019.9310

[75] M. Straczkowski , I. Kowalska , A. Nikolajuk , et al., “Relationship Between Insulin Sensitivity and Sphingomyelin Signaling Pathway in Human Skeletal Muscle,” Diabetes 53 (2004): 1215–1221. 15111489 10.2337/diabetes.53.5.1215

[76] L. K. Hassouneh , O. A. Timofiychuk , and N. A. Babenko , “Acid Sphingomyelinase Inhibitors, Imipramine and Zoledronic Acid, Increase Skeletal Muscle Tissue Sensitivity to Insulin Action at Old Age,” General Physiology and Biophysics 37 (2018): 163–174. 29125130 10.4149/gpb_2017020

[77] E. Somm , S. A. Montandon , U. Loizides‐Mangold , et al., “The GLP‐1R agonist liraglutide Limits Hepatic Lipotoxicity and Inflammatory Response in Mice Fed a Methionine‐Choline Deficient Diet,” Translational Research 227 (2021): 75–88. 32711187 10.1016/j.trsl.2020.07.008

[78] G. Pontarin , P. Ferraro , L. Bee , P. Reichard , and V. Bianchi , “Mammalian Ribonucleotide Reductase Subunit p53R2 is Required for Mitochondrial DNA Replication and DNA Repair In Quiescent Cells,” Proceedings of the National Academy of Sciences of the United States of America 109 (2012): 13302–13307. 22847445 10.1073/pnas.1211289109 PMC3421225

[79] L. Zavileyskiy and V. Bunik , “Regulation of p53 Function by Formation of Non‐Nuclear Heterologous Protein Complexes,” Biomolecules 12 (2022): 327. 35204825 10.3390/biom12020327 PMC8869670

[80] A. Bourdon , L. Minai , V. Serre , et al., “Mutation of RRM2B, Encoding p53‐Controlled Ribonucleotide Reductase (p53R2), Causes Severe Mitochondrial DNA Depletion,” Nature Genetics 39 (2007): 776–780. 17486094 10.1038/ng2040

[81] R. D. Pitceathly , C. Smith , C. Fratter , et al., “Adults With RRM2B‐Related Mitochondrial Disease Have Distinct Clinical and Molecular Characteristics,” Brain 135 (2012): 3392–3403. 23107649 10.1093/brain/aws231 PMC3501970

[82] W. J. Chen , I. H. Lin , C. W. Lee , et al., “Ribonucleotide Reductase M2B in the Myofibers Modulates Stem Cell Fate in Skeletal Muscle,” NPJ Regenerative Medicine 7 (2022): 37. 35906243 10.1038/s41536-022-00231-w PMC9338274

[83] M. Kaller , W. Shi , and H. Hermeking , “c‐MYC‐Induced AP4 Attenuates DREAM‐Mediated Repression by p53,” Cancers (Basel) 15 (2023): 1162. 36831504 10.3390/cancers15041162 PMC9954515

[84] H. L. Ou and B. Schumacher , “DNA Damage Responses and p53 in the Aging Process,” Blood 131 (2018): 488–495. 29141944 10.1182/blood-2017-07-746396 PMC6839964

[85] M. L. Kuo , A. J. Sy , L. Xue , et al., “RRM2B Suppresses Activation of the Oxidative Stress Pathway and is Up‐Regulated by p53 During Senescence,” Scientific Reports 2 (2012): 822. 23139867 10.1038/srep00822 PMC3492868

[86] S. Geng , W. Zhu , S. Wang , et al., “P53 Modulates Hepatic Insulin Sensitivity Through NF‐κB and p38/ERK MAPK Pathways,” Biochemical and Biophysical Research Communications 495 (2018): 2139–2144. 29258820 10.1016/j.bbrc.2017.12.085

[87] M. M. Marron , T. B. Harris , R. M. Boudreau , et al., “Metabolites Associated With Vigor to Frailty Among Community‐Dwelling Older Black Men,” Metabolites 9 (2019): 83. 31052232 10.3390/metabo9050083 PMC6572139

[88] Y. Duan , K. Tao , Z. Fang , and Y. Lu , “Possible‐Sarcopenic Screening With Disturbed Plasma Amino Acid Profile in the Elderly,” BMC Geriatrics 23 (2023): 427. 37438737 10.1186/s12877-023-04137-0 PMC10337190

[89] A. H. Lancha, Jr. , M. B. Recco , D. S. Abdalla , and R. Curi , “Effect of Aspartate, Asparagine, and Carnitine Supplementation in the Diet on Metabolism of Skeletal Muscle During a Moderate Exercise,” Physiology & Behavior 57 (1995): 367–371. 7716217 10.1016/0031-9384(94)00243-x

[90] J. M. Hinkley , H. H. Cornnell , R. A. Standley , et al., “Older Adults With Sarcopenia Have Distinct Skeletal Muscle Phosphodiester, Phosphocreatine, and Phospholipid Profiles,” Aging Cell 19 (2020): e13135. 32468656 10.1111/acel.13135 PMC7294783

[91] S. Oveisgharan , T. Wang , L. L. Barnes , J. A. Schneider , D. A. Bennett , and A. S. Buchman , “The time Course of Motor and Cognitive Decline in Older Adults and Their Associations With Brain Pathologies: A Multicohort Study,” Lancet Healthy Longevity 5 (2024): e336–e345. 38582095 10.1016/S2666-7568(24)00033-3 PMC11129202

[92] R. S. Kamper , J. Alcazar , L. L. Andersen , et al., “Associations Between Inflammatory Markers, Body Composition, and Physical Function: The Copenhagen Sarcopenia Study,” Journal of Cachexia, Sarcopenia and Muscle 12 (2021): 1641–1652. 34708570 10.1002/jcsm.12832 PMC8718077

[93] A. E. Davis, 3rd , P. Mejia , and F. Lu , “Biological Activities of C1 Inhibitor,” Molecular Immunology 45 (2008): 4057–4063. 18674818 10.1016/j.molimm.2008.06.028 PMC2626406

[94] S. Zhang , J. Shaw‐Boden , Y. Banz , et al., “Effects of C1 Inhibitor on Endothelial Cell Activation in a Rat Hind Limb Ischemia‐Reperfusion Injury Model,” Journal of Vascular Surgery 68 (2018): 209S–221S.e2. 29395422 10.1016/j.jvs.2017.10.072

