# Supplementary material for: The Impact of SGLT1 Inhibition on Frailty and Sarcopenia: A Mediation Mendelian Randomization Study
Source: J Cachexia Sarcopenia Muscle. 2024 Oct 30;15(6):2693–704. doi: 10.1002/jcsm.13614 (PMC11634476; doi:10.1002/jcsm.13614)
Supplement: Supplementary file 1 — Supporting Information. [file JCSM-15-2693-s001.docx]

**Supporting Information**

**Article title: The Impact of SGLT1 Inhibition on Frailty and Sarcopenia: A Mediation Mendelian Randomization Study**

Journal name: Journal of Cachexia, Sarcopenia and Muscle

Author names: Bang-Bang Huang, MD, Yu-Jie Zhang, PhD, Guang-Feng Ruan, PhD, Xing Yu, MD, Qin Liu, BD^h^, Mei-Jin Zhang, PhD, Ming-Zhong Yu, MD, Ai Chen, PhD, Ye-Bei Liang, PhD, Liang-Di Xie, MD, FESC, FACC, PhD, Li Luo, MD, PhD

# **Supplementary Methods**

**1.1 Methods S1 Instrumental variables**

**1.1.1 Instrumental variables for plasma proteins**

Cis-Protein quantitative trait loci (pQTLs) were meticulously sorted by Zhang et al. to mimic the expression level of plasma proteins^1^, which were utilized as the instrumental variables for plasma proteins. A total of 3811 cis-pQTLs associated with 1558 proteins from 8 cohorts, including KORA F4 study, LBC1936, Fenland study, INTERVAL study, Icelandic Cancer Project and deCODE genetics, IMPROVE study, MANOLIS study, and AGES-Reykjavik study, were ultimately selected^2-9^ (Supplementary Table 1). Of 1588 proteins, 1467 had instrumental variables with complete information in GWAS database of FI, while 1505 out of 1558 proteins possessed instrumental variables having complete information in GWAS database of low grip strength under both FNIH and EWGSOP criteria.

**1.1.2 Instrumental variables for plasma metabolites**

Genetic variants from GWAS statistics of 1352 metabolites in CLSA cohort^10^ was screened to identify the instrumental variants representing the levels of metabolites (Supplementary Table 1). Firstly, SNPs associated with the metabolites levels( *P* < 1×10^-5^) were selected. Then, the selected variants were further clumped by excluding the SNPs in LD of r^2^<0.001 within 10000kb. Finally, weak instrumental variants, defined as F<10, were removed from the analysis (Supplementary Table 3).

**1.1.3 Instrumental variables for insulin resistance phenotype**

In this study, we detected whether insulin resistance played a role in mediating the effect of SGLT1 inhibition on outcomes. A total 53 variants identified in a GWAS database based on 188,577 European was demonstrated to be associated with the insulin resistance phenotype which was characterized by higher fasting insulin levels adjusted for body mass index (BMI), higher triglyceride levels, and lower high-density lipoprotein cholesterol (HDL-C) levels^11^. Because neither β estimates nor standard errors (SEs) were published in this original GWAS, the information of these variants were obtained from a subsequent MR study which utilized meta-analysis to estimate the absolute value of the standardized β coefficient for each of the 53 variants associated with the three components of the insulin resistance phenotype^12^. The data utilized in the study came from MAGIC^13,14^ and GLGC cohort^15^ (Supplementary Table 1). In present study, the insulin resistance referred to 55% higher geometric mean of fasting insulin, 0.89 mmol/L higher triglycerides, and 0.46 mmol/L lower HDL-C^12^.

**1.2 Methods S2 Mediation MR analysis**

**1.2.1 Mediation MR analysis to evaluate the role of insulin resistance in the associations between SGLT1 inhibition and frailty as well as low grip strength**

After removing the outliers identified by MR-PRESSO, the casual relationships between insulin resistance and outcomes were assessed by IVW method (β2). Then the estimated association between SGLT1 inhibition and insulin resistance was examined (β1). Subsequently, the mediation proportion of insulin resistance in the association between SGLT1 inhibition and outcomes was calculated as (β1×β2)/β0. The 95% confidence intervals (95% CI) of the mediation proportions were calculated through the delta method.

**1.2.2 Mediation MR analysis to evaluate the role of plasma proteins in the associations between SGLT1 inhibition and frailty as well as low grip strength.**

Two-step MR analysis was conducted to identify the potential mediators in the relationships between SGLT1 inhibition and outcomes. Initially, the associations between plasma proteins and outcomes were assessed (β2). The MR effects were estimated using the Wald ratio method when only one cis-pQTL was present, and the IVW method when two or more were present^1^. Next, the impact of SGLT1 inhibition on these proteins that has been proved to be associated with outcomes was evaluated (β1). The delta method was then conducted to identify the mediators among those proteins showing significantly associations with both SGLT1 inhibition and outcomes, and calculate the mediation proportions[(β1×β2)/β0] as well as the 95%CIs.

**1.2.3 Mediation MR analysis to evaluate the role of plasma metabolites in the associations between SGLT1 inhibition and frailty as well as low grip strength.**

Firstly, the relationships between plasma metabolite levels and outcomes were assessed through IVW method (β2). Secondly, the effect of SGLT1 inhibition on the metabolites associated with the outcomes was further examined (β1). Finally, the mediators in the associations between the SGLT1 inhibition and outcomes were determined among these selected metabolites and the mediation proportions were calculated[(β1×β2)/β0] by using the delta method.

**1.3 Methods S4 Sensitivity analysis**

The effect of SGLT1 inhibition on low grip strength was further estimated across genders to ascertain the stability of the results. Considering the SGLT1 inhibition was associated with low grip strength in both males and females under FNIH criteria, the potential mediators in the relationships between SGLT1 inhibition and low grip strength across genders were identified.

In addition, three specific protein-altering variants in the SLC5A1 gene, previously reported as representative of the loss-of-function of SGLT1^16,17^, were utilized as instrumental variables in validation analysis to examine the reliability of the study (Supplementary Table 5).

Different GWAS databases of plasma proteins based on various population with European ancestry were used to validate the mediating effects of the identified mediators. Evaluations was performed by assessing the strength of evidence according to the numbers of databases that could replicate the results.

1. **Supplementary references**

1. Zhang Y, Xie J, Wen S, Cao P, Xiao W, Zhu J, et al. Evaluating the causal effect of circulating proteome on the risk of osteoarthritis-related traits. *Ann Rheum Dis* 2023; **82**:1606-1617.

2. Suhre K, Arnold M, Bhagwat AM, Cotton RJ, Engelke R, Raffler J, et al. Connecting genetic risk to disease end points through the human blood plasma proteome. *Nat Commun* 2017; **8**:14357.

3. Hillary RF, McCartney DL, Harris SE, Stevenson AJ, Seeboth A, Zhang Q, et al. Genome and epigenome wide studies of neurological protein biomarkers in the Lothian Birth Cohort 1936. *Nat Commun* 2019; **10**:3160.

4. Pietzner M, Wheeler E, Carrasco-Zanini J, Raffler J, Kerrison ND, Oerton E, et al. Genetic architecture of host proteins involved in SARS-CoV-2 infection. *Nat Commun* 2020; **11**:6397.

5. Sun BB, Maranville JC, Peters JE, Stacey D, Staley JR, Blackshaw J, et al. Genomic atlas of the human plasma proteome. *Nature* 2018; **558**:73-79.

6. Ferkingstad E, Sulem P, Atlason BA, Sveinbjornsson G, Magnusson MI, Styrmisdottir EL, et al. Large-scale integration of the plasma proteome with genetics and disease. *Nat Genet* 2021; **53**:1712-1721.

7. Folkersen L, Fauman E, Sabater-Lleal M, Strawbridge RJ, Frånberg M, Sennblad B, et al. Mapping of 79 loci for 83 plasma protein biomarkers in cardiovascular disease. *PLoS Genet* 2017; **13**:e1006706.

8. Gilly A, Park YC, Png G, Barysenka A, Fischer I, Bjørnland T, et al. Whole-genome sequencing analysis of the cardiometabolic proteome. *Nat Commun* 2020; **11**:6336.

9. Gudjonsson A, Gudmundsdottir V, Axelsson GT, Gudmundsson EF, Jonsson BG, Launer LJ, et al. A genome-wide association study of serum proteins reveals shared loci with common diseases. *Nat Commun* 2022; **13**:480.

10. Chen Y, Lu T, Pettersson-Kymmer U, Stewart ID, Butler-Laporte G, Nakanishi T, et al. Genomic atlas of the plasma metabolome prioritizes metabolites implicated in human diseases. *Nat Genet* 2023; **55**:44-53.

11. Lotta LA, Gulati P, Day FR, Payne F, Ongen H, van de Bunt M, et al. Integrative genomic analysis implicates limited peripheral adipose storage capacity in the pathogenesis of human insulin resistance. *Nat Genet* 2017; **49**:17-26.

12. Wang Q, Holmes MV, Davey Smith G, Ala-Korpela M. Genetic Support for a Causal Role of Insulin Resistance on Circulating Branched-Chain Amino Acids and Inflammation. *Diabetes Care* 2017; **40**:1779-1786.

13. Manning AK, Hivert MF, Scott RA, Grimsby JL, Bouatia-Naji N, Chen H, et al. A genome-wide approach accounting for body mass index identifies genetic variants influencing fasting glycemic traits and insulin resistance. *Nat Genet* 2012; **44**:659-669.

14. Scott RA, Lagou V, Welch RP, Wheeler E, Montasser ME, Luan J, et al. Large-scale association analyses identify new loci influencing glycemic traits and provide insight into the underlying biological pathways. *Nat Genet* 2012; **44**:991-1005.

15. Willer CJ, Schmidt EM, Sengupta S, Peloso GM, Gustafsson S, Kanoni S, et al. Discovery and refinement of loci associated with lipid levels. *Nat Genet* 2013; **45**:1274-1283.

16. Seidelmann SB, Feofanova E, Yu B, Franceschini N, Claggett B, Kuokkanen M, et al. Genetic Variants in SGLT1, Glucose Tolerance, and Cardiometabolic Risk. *J Am Coll Cardiol* 2018; **72**:1763-1773.

17. Peschard S, Raverdy V, Bauvin P, Goutchtat R, Touche V, Derudas B, et al. Genetic Evidence of Causal Relation Between Intestinal Glucose Absorption and Early Postprandial Glucose Response: A Mendelian Randomization Study. *Diabetes* 2024; **73**:983-992.
